# Abrasions and lameness in piglets born in different farrowing systems with different types of floor

**DOI:** 10.1186/1751-0147-50-37

**Published:** 2008-09-25

**Authors:** Mate Zoric, Ebba Nilsson, Sigbrit Mattsson, Nils Lundeheim, Per Wallgren

**Affiliations:** 1 National Veterinary Institute, SVA, 751 89 Uppsala, Sweden; 2 Department of Clinical Sciences, Swedish University of Agricultural Sciences, SLU, 750 07 Uppsala, Sweden; 3 Department of Animal Breeding and Genetics, Swedish University of Agricultural Sciences, Box 7023, S-750 07 Uppsala, Sweden

## Abstract

**Background:**

The quality of the floor is essential to the welfare of piglets as abrasions often are recorded in newborn piglets, and such lesions may lead to lameness. Apart from animal suffering, lameness contributes to losses in form of dead piglets, decreased growth, and increased use of antibiotics and manual labour.

**Methods:**

In a herd with three different farrowing systems, 37 litters (390 piglets) were studied until the age of 3 weeks with respect to presence of skin wounds and abrasions. Lameness was registered until the age of 7 weeks. Eight lame piglets were sacrificed before medical treatment and subjected to necropsy including histopathological and microbiological examinations. Isolates of streptococci, staphylococci and *E. coli *were tested with respect to antimicrobial resistance. Mastitis was observed in ten sows.

**Results:**

The most severe abrasions at carpus and soles were seen in the system with a new solid concrete floor with a slatted floor over the dunging area. The lowest magnitude was observed in the deep litter system with peat. Sole bruising was more common in the systems with concrete floor compared to the deep litter system with peat, and the differce in prevalence was significant at all examination days. The lesions decreased with time and about 75% of the treatments for lameness were performed during the first three weeks of life. The overall prevalence of lameness was highest in the system with new solid concrete floor with a slatted floor over the dunging area (9.4%) followed by the old solid concrete floor (7.5%). A lower (p < 0.05) prevalence was seen in the deep litters system with peat (3.3%). No significant relationship between mastitis and abrasions or lameness in the offspring was observed.

**Conclusion:**

There were large differences in the prevalence of abrasions and lameness between the floor types. The deep litter system with peat provided a soft and good floor for piglets. The overall prevalence of lameness was only diagnosed in every fourth litter in that system compared to in every second litter in the systems with concrete floor. In contrast, the incidence of mastitis in the sows during the first week after farrowing was higher than in the systems with concrete floor.

## Background

Abrasions, wounds and necrosis in the skin or on the hooves and accessory digits, are very common in newborn piglets, and can almost be classified as "normal" in most pig farms [1,2]. Foot and skin lesions cause discomfort to the piglets and also provide an entry for infections, which may result in lameness [1]. Lameness in sucking piglets is observed in about every second litter and around 75% of the treatments against lameness are effectuated in very young piglets, less than 3 weeks of age [3-5].

Joint swelling and lameness are the most obvious and persistent clinical signs of infectious arthritis. Piglets can be infected with different pathogenic or facultative pathogenic microorganisms either directly from the sow and/or via the environment. Invading bacteria may enter the bloodstream via skin wounds, the navel or the tonsils [6]. In lame piglets under 12 weeks of age the causative agents of arthritis have been reported to include *Streptococcus dysgalactiae *subsp. *equisimilis *(26.3%), *Staphylococcus hyicus *(24.6%), *Arcanobacterium pyogenes *(13.2%), *S. aureus *(7.9%), and *Haemophilus parasuis *(7.9%), and most of the pigs culled for arthritis were under 6 weeks of age [7]. The streptococci domination suggests the sow to be a significant source of infection to the piglets [8].

Risk factors associated with foot and skin lesions in all age groups of pigs include floor type, nutrition and genetics [1]. The skin lesions in piglets are presumably mainly a result of contact with the floor, especially during suckling [9,10]. The lesions are generally bilateral and most commonly observed as abrasions over the carpal joints. They are present already on day 3, increase in magnitude until day 10 and thereafter decline [5].

The quality of the floor is essential to welfare and is likely to have a direct effect on foot health and the culling rate from lameness. Concrete floors can be rough and abrasive, thereby contributing to removal of horn from hooves and skin from especially the limbs in newly born piglets which may cause acute lameness [11]. Piglets kept on bare concrete or slats can not carry out their natural foraging and exploring behaviour, while piglets kept on peat and straw are more active and less aggressive [12]. Peat has historically been important as litter in the animal production, but the use of peat as litter within Swedish agriculture reached its peak during the 1930s. Then the use of peat litter almost disappeared entirely, and thereby also the knowledge about peat as litter vanished [13]. However, a new farrowing pen with deep litter peat that housed piglets born until the age of 12 weeks was designed in the 1990ies by a farmer (Jan Vallgårda, Vallrum, Sweden).

Proper care and attention to the floor in farrowing pens is essential to reduce the severity of the problems associated to lameness in piglets. The present study scrutinised the effect of floor on the incidence and severity of abrasions, as well as, on the incidence of lameness, in nursing piglets in three different farrowing pen systems within one herd.

## Methods

### Herd, animals and management routines

The study was performed in a conventional farrow to finish farm with 250 Yorkshire × Landrace sows in production. While pregnant, the sows were housed in a deep-litter straw system in groups of 40 to 45 animals, but were fed individually in stalls. Three days before expected farrowing, the sows were transferred to farrowing units with individual farrowing pens. There were 3 units with different types of farrowing pens in the herd, all managed by the same staff: an old housing system, with 21 farrowing pens, a new housing system with 24 farrowing pens and a deep litter peat housing system with 40 farrowing pens.

After farrowing, the canine teeth of the piglets were filed. The male piglets were castrated at three to five days of age and at the same time all the piglets received an intramuscular injection of 200 mg iron as iron dextran (Pigeron: Leo Pharmaceutical, Copenhagen, Denmark). The piglets were given a second iron injection ten days later. The piglets were offered a commercial creep feed without antibiotics from the age of ten days, and they were weaned at an average age of 4.5 weeks.

Every medically treated piglet was colour-marked, and records of diseases and treatments were kept for each sow and piglet. The veterinarian of the herd had given instructions for diagnosing and decided what treatment was to be given, but the staff of the herd generally effectuated them.

### Experimental design

This study was carried out in a conventional herd with three different farrowing systems managed by the same staff, and as earlier observations had revealed the parity number not influenced lameness in the offspring [4] we focused to create as large groups as possible that were born on the same day. The study included three consecutive farrowing batches; *i.e*. one farrowing batch within each of the 3 farrowing systems:

1) The old farrowing system with solid concrete floor had pens of 5.9 m^2 ^including the dunging area. Of this, the creeping area for the piglets was 0.7 m^2^. The floor was old and embedded with chopped straw (n = 11 sows with 120 live born piglets, mean parity 4.4 ± 2.2).

2) The new farrowing system had pens with 5.7 m^2 ^concrete floor out of which 0.9 m^2 ^was a creeping area for the piglets and 1.7 m^2 ^slatted floor (dunging area). The floor was new and embedded with chopped straw (n = 11 sows with 117 live born piglets, mean parity 2.5 ± 1.3).

3) The farrowing system with deep litter peat had pens of 5.6 m^2^, of which 0.7 m^2 ^was a creeping area for the piglets (n = 15 sows with 153 live born piglets, mean parity 4.0 ± 1.4).

Straw was provided at a rate of approximately 5 kg/week and pen in the systems with solid concrete floor. In all farrowing pens, the creep area was heated with infrared lamps.

### Examination of feet, limbs and skin in piglets

The piglets were individually examined day 3, day 10 and day 17. They were restrained and examined for presence of skin lesions of the carpus, hock, abdomen and teats, face and tail. The feet were examined with respect to presence of sole bruising. Sole bruising was defined as congestion and bruising of the solar corium presenting as a dark red pigmentation on the volar surface of the foot. The sex of each piglet was recorded and the healing of castration wounds was examined the 10^th ^and 17^th ^day of age. All examinations were performed by one and the same veterinarian. The severity of the lesions was scored and examined by using protocols previously described [5] as seen in table 1.

**Table 1:** The severity of the lesions recorded at day 3, day 10, day 17 was scored as 0, 1, 2 or 3 defined as shown below.

**Score**	**Skin lesions**	**Sole bruising/Sole erosion**	**Castration wounds**
**0 – No lesion**	-	-	-
**1 – Mild**	Hairless patches or loss of hair and mild hyperkeratosis	Small part of the volar surface of digit affected	Mild inflammation, eczema or oedema
**2 – Moderate**	Skin abrasions	Less than half of the volar surface of the digit affected	Clinical signs of inflammation, swelling, redness, localized warmth
**3 – Severe**	Skin wounds. Spots of induration or scab that is a hard mass mainly of dried blood.	More than half of the volar surface digit affected or sole erosion, loss of horny tissue.	Inflamed castration wounds with purulence. Smelly wound. Abscess.

### Lameness, necropsies, culture of bacteria, antimicrobial resistance

If affected by arthritis, piglets have to be treated during an early phase of the infection to attain a fair prognosis. Therefore, the herd veterinarian had made a written instruction to the staff. According to that instruction, lame piglets or piglets with visibly swollen joints were defined to suffer from arthritis and should be parenterally treated with antimicrobials.

Eight randomly selected lame piglets (3 from each of the solid floor systems and 2 from the peat system) were sacrificed instead of medically treated. To exclude sprains, these piglets had to be lame and/or express visibly swollen joint(s) for 2 days before the diagnosis was given. Their carcasses were stored in -20°C until necropsied. Samples for bacteriology were collected with sterile cotton swabs from up to 3 affected joints and from a normal joint from each pig at necropsy. The samples were spread directly to blood agar (blood agar base No. 2; LabM, Salford, England + 5% horse blood) and bromcresol purple-lactose agar (NVI art No.341200). The plates were incubated at 37°C and read after 18 and 48 hours. Isolates of staphylococci, streptococci and *E. coli *were typed with methods used at the Bacteriological diagnostic laboratory at the National Veterinary Institute (NVI).

Isolates of staphylococci and streptococci were tested with respect to antimicrobial resistance towards penicillin, ampicillin, ceftiofur, spiramycin, neomycin, gentamicin, streptomycin, trimethoprim/sulfametoxazol, enrofloxacin, oxytetracycline, florfenicol and oxacillin (VetMIC™ Large Animal, NVI). Isolates of *E. coli *were tested for antimicrobial resistance towards ampicillin, ciprofloxacin, nalidixic acid, gentamicin, ceftiofur, streptomycin, tetracycline, florfenicol, kanamycin, sulfamethoxazole, trimethoprim, chloramphenicol and cefotaxime (VetMIC™ GN-mo, NVI).

### Mastitis in sows

Mastitis was defined as fever, loss of appetite and inflamed udder and hypogalactia. Sows with mastitis were parenterally treated with 15 mg trimethoprim + sulphonamide per kilogram body weight for five consecutive days.

### Statistical analyses

Data from the 390 piglets from the 37 litters from three different farrowing pen systems were included in the study. The prevalence of findings at each inspection (day 3, day 10, day 17) was calculated as the number of piglets with that lesion divided with the number of piglets examined at that occasion.

Only the first time score for treatment due to lameness was included in the statistical analyses. Thus recurrence of lameness was ignored in the calculations. The weekly risk incidence for treatment due to lameness was calculated as the number of piglets affected by lameness during the actual period divided by the number of live piglets previously not affected by lameness at the beginning of that week.

Preweaning mortality was calculated as number of dead piglets (mainly by crushing) divided by the number of live born piglets.

Data was statistically analysed using the SAS software ver. 9 (SAS Inst. Inc., Cary, NC, USA). Mean values of the piglets skin lesion scores and castration wounds (scores 0 to 3) were calculated for each housing system – sow – inspection day – sex-combination. These mean values were further analysed by analysis of variance using PROC MIXED. The statistical model included the fixed effects of housing system (3), day (3), sex (2) and the interaction between system and day. Also, the random effect of sow, nested within housing system was included in the statistical model. In the statistical model for analysing castration wounds, the effect of sex was excluded. Least-squares means were calculated for each level of the fixed effects, and pairwise tests of significance were performed using t-test.

## Results

**Figure 1:**
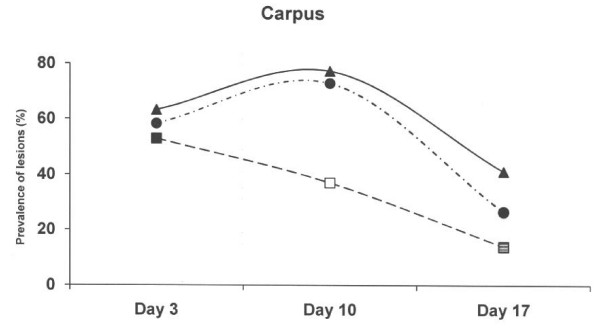
**Prevalence of skin lesions scored as 1–3 at carpus in three different farrowing pen systems.** Circles represent an old solid concrete floor embedded with chopped straw, triangles represent a new solid concrete floor with a slatted floor over the dunging area embedded with chopped straw and squares represent a deep litters system with peat. Symbols other than black represent a statistic difference to black within examination day (shade = p < 0.05; grey = p < 0.01; white = p < 0.001).

The skin lesions were generally bilateral and most commonly observed as abrasions over the carpal joints (Figure 1, Table 3). On day 3, the skin lesions at carpus were dominated by mild to moderate lesions in all three farrowing pen systems (n = 117, 63% in the new concrete floor with slatted floor over the dunging area; n = 120, 58% in the old solid concrete floor and; n = 153, 53% in the deep litter peat system). In the concrete floor systems, these lesions increased in prevalences and magnitudes until day 10, but decreased at day 17 (via 77% to 41% in the new concrete floor with slatted floor over the dunging area; via 73% to 27% in the old solid concrete floor). In contrast, the prevalence of the carpal lesions decreased significantly with time in the deep litter peat system (37% day 10; p < 0.001 and 14% day 17; p < 0.05).

**Figure 2:**
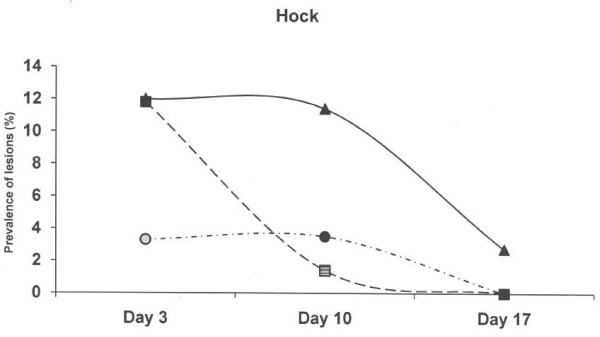
**Prevalence of skin lesions scored as 1–3 at the hocks in three different farrowing pen systems.** Circles represent an old solid concrete floor embedded with chopped straw, triangles represent a new solid concrete floor with a slatted floor over the dunging area embedded with chopped straw and squares represent a deep litters system with peat. Symbols other than black represent a statistic difference to black within examination day (shade = p < 0.05; grey = p < 0.01; white = p < 0.001).

**Figure 3:**
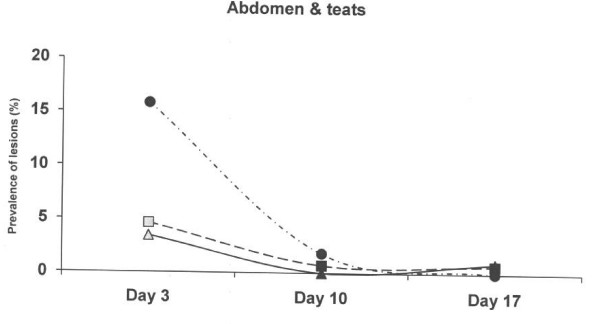
**Prevalence of skin lesions scored as 1–3 at abdomen and teats in three different farrowing pen systems.** Circles represent an old solid concrete floor embedded with chopped straw, triangles represent a new solid concrete floor with a slatted floor over the dunging area embedded with chopped straw and squares represent a deep litters system with peat. Symbols other than black represent a statistic difference to black within examination day (shade = p < 0.05; grey = p < 0.01; white = p < 0.001).

**Figure 4:**
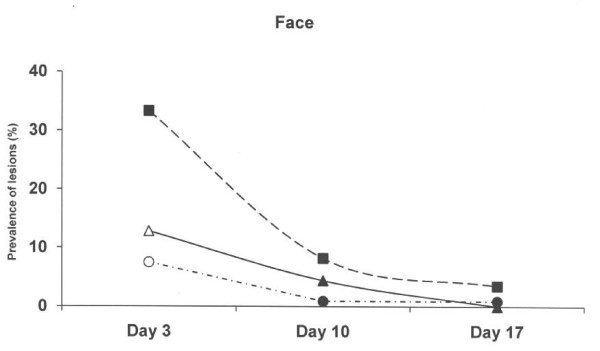
**Prevalence of skin lesions scored as 1–3 in the face in three different farrowing pen systems.** Circles represent an old solid concrete floor embedded with chopped straw, triangles represent a new solid concrete floor with a slatted floor over the dunging area embedded with chopped straw and squares represent a deep litters system with peat. Symbols other than black represent a statistic difference to black within examination day (shade = p < 0.05; grey = p < 0.01; white = p < 0.001).

**Figure 5:**
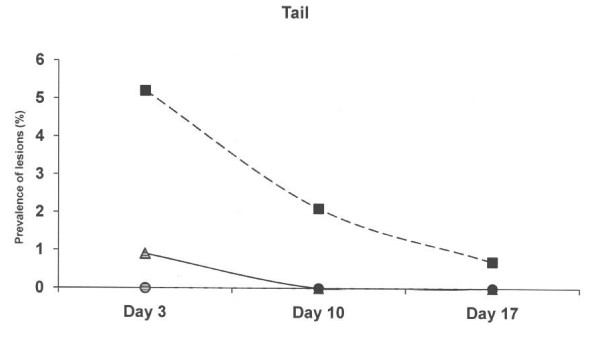
**Prevalence of skin lesions scored as 1–3 at tail in three different farrowing pen systems.** Circles represent an old solid concrete floor embedded with chopped straw, triangles represent a new solid concrete floor with a slatted floor over the dunging area embedded with chopped straw and squares represent a deep litters system with peat. Symbols other than black represent a statistic difference to black within examination day (shade = p < 0.05; grey = p < 0.01; white = p < 0.001).

At 3 days of age, piglets born on the old solid concrete floor expressed a lower (p < 0.01) prevalence of lesions at the hocks compared to piglets born on the new concrete floor with a slatted floor over the dunging area and the deep litter system (Figure 2), but a higher (p < 0.01) prevalence of abrasion at abdomen and teats (Figure 3). Skin lesions at the hocks and the abdominal & teat abrasions decreased from day 3 to day 10, and had practically vanished on day 17 in all three farrowing pen systems. The prevalence of skin lesions in the face (p < 0.001) and tail (p < 0.05) were significantly higher in the deep litter peat system than in the other systems when the piglets were aged 3 days, but not later (Figure 4 and Figure 5).

**Figure 6:**
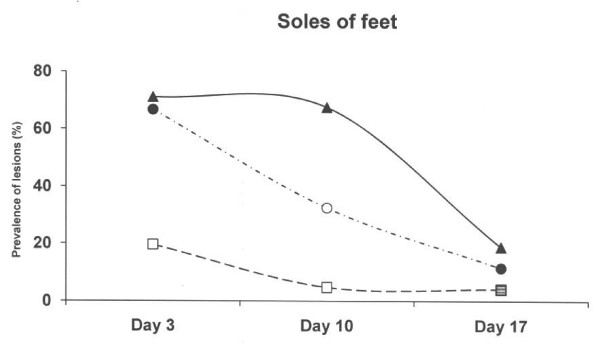
**Prevalence of sole bruising of feet scored as 1–3 in three different farrowing pen systems.** Circles represent an old solid concrete floor embedded with chopped straw, triangles represent a new solid concrete floor with a slatted floor over the dunging area embedded with chopped straw and squares represent a deep litters system with peat. Symbols other than black represent a statistic difference to black within examination day (shade = p < 0.05; grey = p < 0.01; white = p < 0.001).

Sole bruisings were more commonly observed in the systems with concrete floor and both these systems differed significantly from the deep litter peat system (Figure 6). The most severe abrasions at soles were seen in piglets kept on the new solid concrete floor with a slatted floor over the dunging area (Table 3).

Healing of castrations wounds were examined the 10^th ^and 17^th ^day of age. At day 10, mild inflammations were recorded in the meagre half (48%) of the piglets in the old solid concrete floor system, which differed from the new concrete floor system (18%) and the deep litter peat system (15%). At day 17, healthy healing processes without inflammation, eczema or oedema were generally seen in all three farrowing pen systems (Table 3), but inflamed castration wounds with purulence were observed in 3 piglets in the old solid concrete floor system.

**Figure 7:**
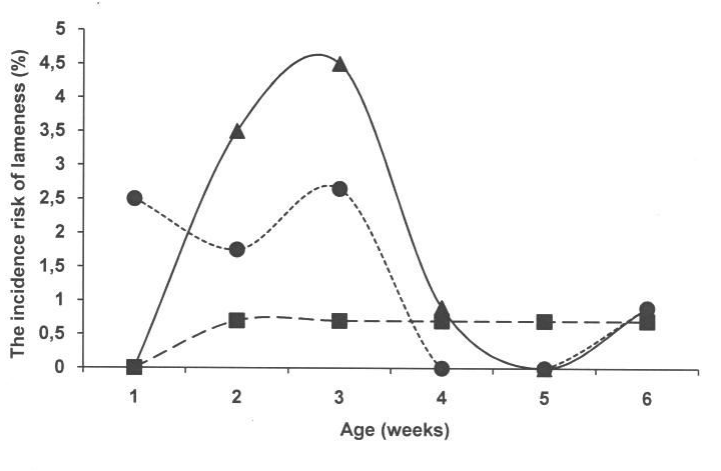
**The incidence risk to be attended for lameness with respect to week of age in all three systems.** Circles represent an old solid concrete floor embedded with chopped straw, triangles represent a new solid concrete floor with a slatted floor over the dunging area embedded with chopped straw and squares represent a deep litters system with peat.

Around 75% of the treatments for lameness were performed during first three weeks of life in the two farrowing systems with concrete floor (Figure 7). The overall prevalence of lameness was highest in the system with a new concrete floor with slatted floor over the dunging area (11 out of 117; 9.4%) followed by the old solid floor (9 out of 120; 7.5%). A lower prevalence (5 out of 153; 3.3%; p < 0.05) was seen in the deep litters system with peat. Lameness was diagnosed in about every second litter in the systems with concrete floor and in about every fourth litter in the deep system with litter peat. The number of affected piglets in affected litters ranged from 1 to 3 (Table 2). In contrast, the number of piglets crushed under the sow was non-significantly higher (5.9%) in the deep litter system with peat than in the farrowing pens with new (4.3%) and old (2.5%) solid concrete floors.

**Table 2:** Numbers of piglets diagnosed as lame until the age of 7 weeks in relation to litters in three different farrowing systems with respect to pen design and type of floor.

			Lame per litter		
			
Floor in farrowing pen	n	%	One piglet	Two piglets	Three piglets
*Old concrete, solid floor*					
Litters with lame piglets	6	55%	4	1	1
Litters with no lame piglets	5				
					
*New concrete, slatted dunging area*					
Litters with lame piglets	7	64%	4	2	1
Litters with no lame piglets	4				
					
*Deep litter system, peat*					
Litters with lame piglets	4	27%	3	1	-
Litters with no lame piglets	11				

**Table 3:** Least-squares means for lesion scores in piglets reared in three different farrowing systems with respect to pen design and type of floor over time.

	Type of floor		
	
	Old concrete, solid floor	New concrete, slatted dunging area	Deep litter system with peat
***Site***	**Day 3**		
	
Carpus	0.9 ± 0.6	1.0 ± 0.5	0.9 ± 0.5
Hock	0.1 ± 0.1	0.1 ± 0.2	0.2 ± 0.2
Abdomen & teats	0.2 ± 0.2	0.0 ± 0.1	0.1 ± 0.1
Face	0.1 ± 0.1	0.1 ± 0.1	0.4 ± 0.3
Tail	0.0 ± 0.0	0.0 ± 0.1	0.1 ± 0.1
Soles of feet	0.9 ± 0.4	1.0 ± 0.5	0.3 ± 0.3
Castration wounds	-	-	-
			
***Site***	**Day 10**		
	
Carpus	1.2 ± 0.5 *	1.3 ± 0.3 ns	0.5 ± 0.3 ***
Hock	0.0 ± 0.1 ns	0.2 ± 0.3 ns	0.0 ± 0.1 ***
Abdomen & teats	0.0 ± 0.1 ***	0.0 ± 0.0 ns	0.0 ± 0.0 **
Face	0.0 ± 0.1 ns	0.0 ± 0.1 ns	0.1 ± 0.1 ***
Tail	0.0 ± 0.0 ns	0.0 ± 0.0 ns	0.1 ± 0.1 ns
Soles of feet	0.4 ± 0.4 ***	0.9 ± 0.3 ns	0.0 ± 0.1 **
Castration wounds	0.7 ± 0.4	0.2 ± 0.2	0.2 ± 0.4
			
***Site***	**Day 17**		
	
Carpus	0.3 ± 0.2 ***	0.5 ± 0.3 ***	0.1 ± 0.2 ***
Hock	0.0 ± 0.0 ns	0.0 ± 0.0 *	0.0 ± 0.0 ns
Abdomen & teats	0.0 ± 0.0 ns	0.0 ± 0.0 ns	0.0 ± 0.1 ns
Face	0.0 ± 0.0 ns	0.0 ± 0.0 ns	0.0 ± 0.0 ns
Tail	0.0 ± 0.0 ns	0.0 ± 0.0 ns	0.0 ± 0.1 ns
Soles of feet	0.2 ± 0.2 *	0.2 ± 0.3 ***	0.0 ± 0.1 ns
Castration wounds	0.2 ± 0.3 ***	0.1 ± 0.2 ns	0.1 ± 0.3 ns

ns, not significant; * p < 0.05; ** p < 0.01; *** p < 0.001;

Statistical remarks indicate comparisons to the score at the previous recording at that actual site within pen category. For differences between the pen categories, see the figures

Eight lame piglets were subjected to necropsy including histopathological and microbiological examinations. A fracture of the left humerus was the main diagnose in one pig, but a fluid accumulation was also seen in both elbows of that pig. The other seven pigs suffered from acute fibrin-purulent arthritis, and 5 of the piglets were affected in more than one joint. Bacterial cultivations of three joints per animal demonstrated microbial growth in all piglets. The findings were *Streptococcus dysgalactiae *subsp. *equisimilis *in 5 piglets (9/24 joints), *Staphylococcus hyicus *subsp.*hyicus *in 5 piglets (6/24 joints) and *E. coli *in 2 piglets (2/24 joints). Different bacterial growths were recorded in four piglets, two piglets with *Streptococcus dysgalactiae *subsp. *equisimilis *and *Staphylococcus hyicus *subsp.*hyicus *and two piglets with *Staphylococcus hyicus *subsp. *hyicus *and *E. coli*, respectively.

No antimicrobial resistance was recorded. The isolates of *Streptococcus dysgalactiae *subsp. *equisimilis *and *Staphylococcus hyicus *subsp.*hyicus *isolates were sensitive to all antibiotics included in the VetMIC™ Large Animal panel, and the isolates of *E. coli *were sensitive to all antibiotics included in the VetMIC™ GN-mo panel.

Mastitis was observed in four sows (27%) in the deep litter system with peat, in two sows (18%) in the old solid concrete floor and one sow (9%) on new concrete floor with slatted floor over the dunging area between the first and the third day after farrowing. During the second week after farrowing, mastitis was observed in the new and old solid concrete floor in the farrowing pens (9% versus 18%), but not in the deep litter system. No correlations between mastitis and lameness or crushed piglets were recorded.

## Discussion

Foot and skin lesions can cause lameness at piglets, either because of pain due to the injury itself or acting as an entrance for infections affecting joints and thereby causing pain [1,5]. If inducing bacteraemia, arthritis, endocarditis, or meningitis may develop [6,14]. As all piglets were handled by the same staff, the results of this study obviously suggest that the immediate environment plays a primary etiological role in the appearance and development of abrasions and lameness in suckling piglets. The explanation is to find in the damage potential of the floor, which depends on the contact surface and the resistance of the piglet. If the force in a strain exceeds the resistance of the skin a physical damage will arise [15,16]. Concrete floors cause more foot and leg problems than earthen floors or deep straw bedding, and injuries occur more frequently on perforated floors. Further, perforated floors such as partially slatted or fully slatted concrete or metal slats cause more lameness than solid concrete floors [17].

In this study, the most severe abrasions at carpus and soles were seen in the system with a new solid concrete floor with a slatted floor over the dunging area. In contrast, the prevalences of abrasion at abdomen, teats and of castration wounds were highest in the system with an old concrete floor. This system had a solid concrete floor in the dunging area, possibly indicating a dirtier environment in that system. Draining floors facilitate removal of dung and thereby potentially decrease the pathogen load, and an increased proportion of the draining floor over the dunging area is generally considered to promote the box hygiene [18].

The skin lesion at carpus and sole bruising were milder in the deep litter system with peat, and so was the incidence of lameness among the piglets. Consequently, the results of this study indicate that a deep litter system with peat provides a soft and good floor for piglets.

However, at day 3 the prevalence of skin lesions in faces and tails was higher in the deep litter peat system than in the systems with new and old solid concrete floors. We have no clear explanation for this, but it may mirror an initial antagonism between the newborn piglets due to difficulties to move in the soft peat. Defending/fighting for a nipple may be beneficial. This phenomenon must however be further studied before any statement are given. As the temperature is higher in the deep litter system with peat than in systems with solid concrete floors under equal weather conditions [13], piglets in the deep litter peat system will apply less for the heat lamp and their special lying scope. Thereby they will expose themselves to an increased risk to be crushed by the sow, and the observed non-significantly higher prevalence of piglets crushed under the sow in this system should not be neglected.

In concordance with earlier studies [13] the overall prevalence of lameness was decreased in the deep litter system with peat where lameness only was diagnosed in every fourth litter. In contrast, lameness was recorded in every second litter in the two systems with solid concrete floors. Lameness in piglets is reported to occur more frequently in farrowing pens with slatted floor without access straw than in pens with solid floors embedded with straw [19]. However, we only recorded a slight and non-significant higher prevalence of lameness in the system with a new concrete floor with a slatted floor over the dunging area compared to the old solid concrete floor. As both these systems had access to an equal amount of straw in our study, the somewhat higher prevalence of lameness in the system with new concrete floor with a slatted floor over the dunging area most likely was an effect of a rougher surface of the new floor rather than of the slatted part of the pen. Indeed the plastic slatted floor over the dunging area probably decreased the exposure of the piglets to microbes of intestinal origin [18].

In deep litter systems, constipation of sows due to peat consumption has frequently been recorded, but not feasible linked to the increased incidence of mastitis [13]. Still, the incidence of mastitis close to farrowing was highest in this group. As affected sows may protect their udder by a sternal position, this may influence the well-being of the offspring. No significant relationship between the health status of the sows and abrasions or lameness in the offspring, nor of crushing, was however observed.

The microbial cause of lameness in piglets may vary and treatment of lame pigs leads to a permanent use of antibiotics in farrowing pens, which in turn may lead to antimicrobial resistance. Therefore, a causative diagnose, including defining of minimum inhibitory concentration (MIC) values, ought to regularly be made from joints of lame piglets in pig herds. In this herd, bacterial cultivations revealed *Streptococcus dysgalactiae *subsp. *equisimilis *as the dominating cause of infectious arthritis followed by *Staphylococcus hyicus *subsp. *hyicus *and *E. coli *which concur with several other reports [20,21]. These isolates were sensitive to all antibiotics included in the antimicrobial panels. However, the importance of regular cultivations was elucidated by the presence of *E. coli *in a few joints, being a gram negative coccus *E. coli *generally differ in MIC-values from the other more commonly isolated microbes. In this context, it was of specific interest that two different microbes were demonstrated in 4 of the 8 piglets.
